# Effect of Enhanced Thermal Stability of Alumina Support Layer on Growth of Vertically Aligned Single-Walled Carbon Nanotubes and Their Application in Nanofiltration Membranes

**DOI:** 10.1186/s11671-018-2585-3

**Published:** 2018-06-07

**Authors:** Jung Bin In, Kang Rae Cho, Tung Xuan Tran, Seok-Min Kim, Yinmin Wang, Costas P. Grigoropoulos, Aleksandr Noy, Francesco Fornasiero

**Affiliations:** 1 School of Mechanical Engineering, Chung-Ang University, Seoul, 156-756 Republic of Korea; 2 The Molecular Foundry, Lawrence Berkeley National Laboratory, Berkeley, CA 94720 USA; 3 Physical and Life Sciences Directorate, Lawrence Livermore National Laboratory, Livermore, CA 94550 USA; 4 Department of Mechanical Engineering, University of California, Berkeley, CA 94720-1740 USA; 5 School of Natural Sciences, University of California, Merced, CA 94343 USA

**Keywords:** Single-walled carbon nanotube, Catalyst, Alumina, CNT membrane

## Abstract

We investigate the thermal stability of alumina supporting layers sputtered at different conditions and its effect on the growth of aligned single-walled carbon nanotube arrays. Radio frequency magnetron sputtering of alumina under oxygen–argon atmosphere produces a Si-rich alumina alloy film on a silicon substrate. Atomic force microscopy on the annealed catalysts reveals that Si-rich alumina films are more stable than alumina layers with low Si content at the elevated temperatures at which the growth of single-walled carbon nanotubes is initiated. The enhanced thermal stability of the Si-rich alumina layer results in a narrower (< 2.2 nm) diameter distribution of the single-walled carbon nanotubes. Thanks to the smaller diameters of their nanotube pores, membranes fabricated with vertically aligned nanotubes grown on the stable layers display improved ion selectivity.

**Electronic supplementary material:**

The online version of this article (10.1186/s11671-018-2585-3) contains supplementary material, which is available to authorized users.

## Background

Single-walled carbon nanotubes (SWCNTs) are promising materials for high-strength composites [1–3], high-speed transistors, flexible electronics [4], and nanofiltration membranes [5–7]. For the latter application, the atomically smooth inner walls of pristine SWNTs provide nearly frictionless channels for molecular transport at extraordinarily fast rates [5, 8]. Tight control on the SWCNT diameter distribution and density is critical to the production of membranes that fully exploit their outstanding fluidic properties and combine high flux with high selectivity and a sharp molecular weight cutoff [9].

Chemical vapor deposition (CVD) has been widely accepted as a controllable and large-scale synthesis method for carbon nanomaterials [10, 11]. Nanoparticles of transition metals such as iron, nickel, and cobalt have been employed in CVD to provide confined catalytic domains necessary for the growth of SWCNTs. If the density of catalyst particles is high enough, SWCNTs self-assemble during growth in vertically aligned arrays (here indicated as VA-SWCNTs), a form that is of particular interest for the fabrication of membranes with highly oriented through pores [5, 6, 12]. Carbon nanotube growth by CVD, however, occurs at high temperatures (500–900 °C in general) where atomic diffusion and the subsequent catalyst ripening processes are significantly accelerated. This thermally induced morphological evolution of catalyst particles can result in reduced catalyst lifetime [13] as well as enlarged nanotube diameters [14].

Not only the thermal stability of a catalyst particle but also the catalyst–substrate interaction is a crucial factor that determines catalyst thermal stability [15]. In this regard, various chemically inert and thermally stable oxide catalyst-support layers such as oxides of silicon [15], aluminum [15, 16], magnesium [17], and zirconium have been examined. In particular, alumina (Al_2_O_3_) thin films have been widely used as a catalyst-support layer for growth of SWCNTs and have been shown to improve the growth yield of SWCNTs (including VA-SWCNTs) by preventing the formation of unwanted metal compounds and improving the dispersion of catalyst nanoparticles [13, 16].

Previous investigations also revealed that the performance of alumina films as a supporting layer for nanotube growth depends on the deposition method. In particular, sputtering was shown to be superior to other thin film deposition methods such as electron-beam evaporation and atomic layer deposition [16, 18]. Researchers have argued that the chemical identity of the alumina film could play a role in such enhanced growth of SWCNTs. This finding naturally opened questions on the importance of alumina stoichiometry and the presence of impurities possibly incorporated into the film during the deposition process [18–20].

In this study, we explored the influence of alumina films sputtered at two different conditions on the growth of VA-SWCNTs at a high temperature (850 °C), where alumina thermal stability becomes critical. To enhance the thermal stability of alumina films, we used a reactive sputtering method (O_2_ + Ar) with a ceramic alumina target [21]. The chemical composition of the alumina film and the morphological change by thermal treatment were scrutinized. We then fabricated nanofiltration membranes from the VA-SWCNTs produced on alumina support layers with different thermal stability and compared their ion selectivity.

## Methods

### Preparation of Alumina and Fe/Mo Catalyst Layers

Radio frequency (RF) sputtering (Edwards Auto 306 DC and RF Sputter Coater) of an alumina target (99.99% pure, Plasmaterials, Inc.) was employed to deposit an alumina film on a substrate. To prevent excessive heating, the alumina target was bonded to an oxygen-free electronic (OFE) copper backing plate. For substrates, p-type silicon wafers (100) with native oxide surfaces were used. Additional heating was not applied to the substrate during the sputtering process.

For nonreactive sputtering, the chamber was pumped out to the base pressure of approximately 3 × 10^−5^ Torr. Before the ignition of plasma, argon gas was introduced, and the pressure reached approximately 5.8 mTorr. Upon the plasma ignition at 210 W (4.8 W/cm^2^), the sputtering process was initiated. The deposition rate was approximately 0.6 nm/min, and the deposition process was finalized when the final thickness of the film became approximately 30 nm. For reactive sputtering, the same procedure was followed, but oxygen gas was additionally introduced and mixed with the argon gas. The presence of oxygen not only increased the chamber process pressure from 5.8 to 6.2 mTorr but also decreased the deposition rate (0.5 nm/min).

To deposit growth catalysts, a very thin Fe/Mo bilayer (0.5 nm/0.2 nm, respectively) was additionally deposited onto the above alumina film by using an e-beam evaporator (Edwards EB3 electron beam evaporator). Fe and Mo targets (99.95–99.99% pure, Plasmaterials Inc.) were used. The base pressure for the catalyst deposition was maintained below 4 × 10^−6^ Torr. After the completion of catalyst deposition, the wafer was cut into individual chips (1 × 1 cm^2^) for the subsequent high-temperature annealing process.

### Alumina Annealing and CVD Growth of VA-SWCNTs

**Fig. 1:**
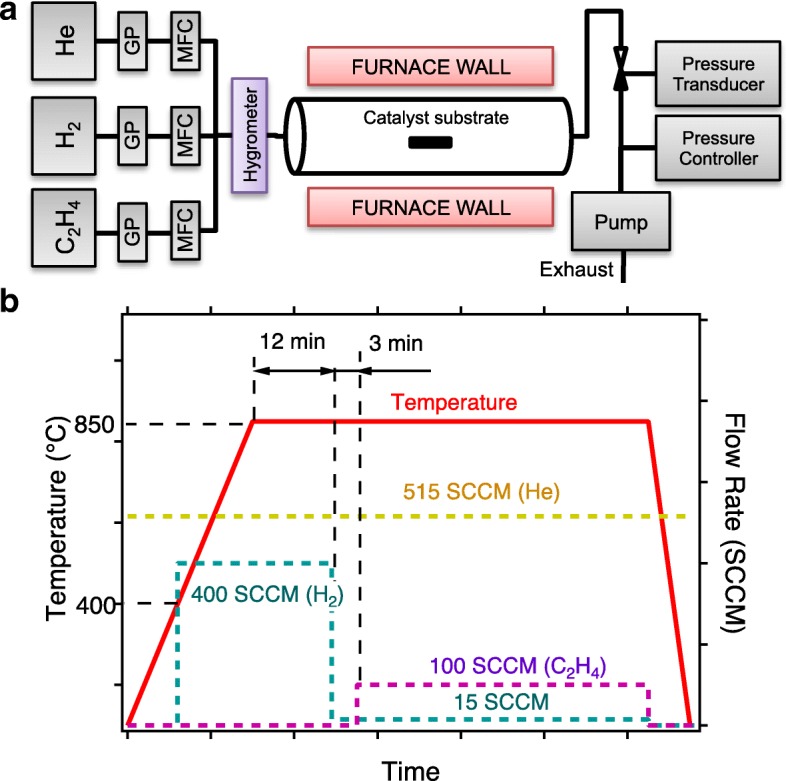
**a** Schematic of the CVD system (GP gas purifier, MFC mass flow controller). **b** CVD process diagram describing the change in furnace temperature and gas combination with respect to process time

For annealing and growth of CNTs at high temperatures, the catalyst sample was placed in home-built atmospheric pressure thermal CVD setup consisting of a gas feeding system and a quartz tube furnace (Lindberg Blue TF55035A, Thermo Electron Corp.) as shown in Fig. 1a. Helium (purity 99.999%, air liquid), hydrogen (purity 99.9999%, air gas), and ethylene (purity 99.999%, air gas) gases were introduced through inline gas purifiers (PureGuard, Johnson Matthey) to the quartz tube. The flow rate of each gas was adjusted using mass flow controllers (MKS). Figure 1b describes the CNT growth process. The catalyst sample was heated to 850 °C at a ramp rate of 50 °C/min. During the temperature ramp, helium (515 SCCM) and hydrogen (at *T* > 400 °C, 400 SCCM) were flown into the quartz tube. The catalyst was then annealed at that temperature under the same gas atmosphere for 12 min. The system was then equilibrated for 3 min at a reduced hydrogen flow rate (15 SCCM). To initiate the growth of CNTs, a gas mixture of ethylene (100 SCCM), hydrogen (15 SCCM), and helium (515 SCCM) was introduced. For annealing-only experiments, the same procedure was followed, but the process was finalized before the introduction of ethylene gas. More details of the growth system and the CVD process can be found in our previous paper [22].

### Characterization of Catalyst Films and Carbon Nanotubes

Surface morphologies of catalyst films were examined using atomic force microscopy (AFM) (MFP 3D, Asylum Research) in a tapping mode. The specimen for cross-sectional transmission electron microscopy (TEM) imaging was prepared by argon ion milling (PIPS691, GATAN). TEM (JEM-ARM200F, JEOL) with energy-dispersive X-ray spectroscopy (EDX) (QUANTAX 400, Bruker) was used for imaging and elemental analysis of the catalyst film. The graphitic structure quality of the as-grown CNTs was evaluated by Raman spectroscopy (Nicolet Almega XR dispersive Raman spectrometer, Thermo Scientific). A HeNe laser (wavelength 632.8 nm) was focused on the top surface of the nanotube arrays through a × 100 objective lens. Laser power was limited to approximately 0.1 mW in order to prevent laser-induced damage on SWCNTs. TEM (Philips CM300-FEG TEM) was also used to obtain a diameter distribution of the nanotubes.

### Fabrication of CNT Membranes and Nanofiltration Experiments

Low-stress silicon nitride (SiN_x_) was conformably deposited by low-pressure chemical vapor deposition (LPCVD) onto as-grown VA-SWCNTs supported by a prepatterned Si wafer. This ceramic material closed the inter-nanotube gaps and provided the CNT membrane with mechanical strength necessary for pressure-driven flow experiments. To open both ends of nanotubes to fluid transport, argon ion milling was first used to remove metal nanoparticles and alumina on the catalyst side, and then, reactive ion etching with oxygen plasma was applied to both sides in order to remove excessive silicon nitride and uncap the nanotubes. The final result was a membrane with VA-SWCNTs as the only through pores in an otherwise impermeable SiN_x_ matrix. A more detailed description of the membrane fabrication process is provided in our previous paper [5]. Representative scanning electron microscopy images (SEM; JEOL7401-F) of CNT membrane cross-sections are given in Fig. 5.

Consistently with previous literature (Additional file 1: Table S1), membranes that showed (a) no macroscopic voids in SEM imaging during the fabrication steps, (b) no detectable flux before etching, (c) enhanced gas and liquid transport rates after opening when compared with classical transport theories, (d) a gas permeance independent of applied pressure, and (e) fully rejected 5-nm gold nanoparticles during filtration were judged to be defect free and then used for ion rejection studies. The filtration cell and protocols for the nanofiltration experiments and capillary electrophoresis (CE) analysis are described in detail elsewhere [5, 6]. Briefly, 2 ml of 1 mM potassium chloride (KCl, 99.999%, Aldrich) or 0.5 mM potassium sulfate solution (K_2_SO_4_, 99%, Sigma, St. Louis, MO) was pressurized at a 0.69-bar pressure differential through a CNT membrane with a controlled nitrogen gas line. After 150–200 μl of solution had permeated through the CNT membrane, samples from both feed and permeate were collected for analysis by capillary electrophoresis (Hewlett Packard 3D CE system, Agilent Technologies, Santa Clara, CA). Ion rejection coefficients were obtained from the CE chromatograms by quantifying the permeate/feed peak–area ratio of the corresponding ion.

## Results and Discussion

### Thermal Stability of Alumina Layer

**Fig. 2:**
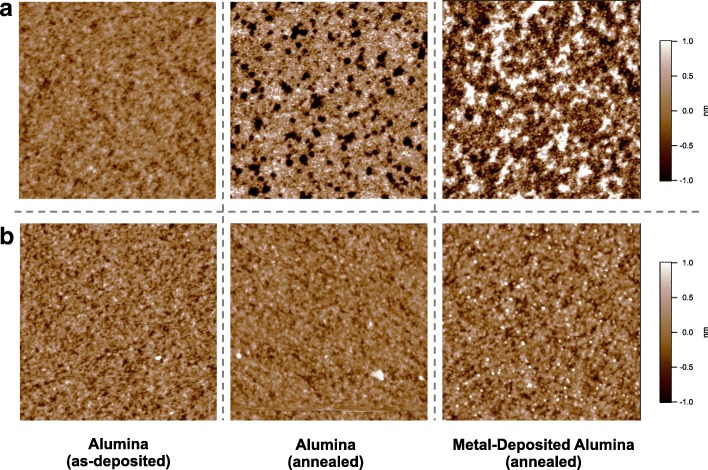
AFM images of alumina and Fe/Mo/alumina catalyst surface that show morphology change by thermal annealing (*T*_a_ = 850 °C.) Alumina was deposited by nonreactive sputtering with argon (**a**) and by reactive sputtering with argon and oxygen (**b**). The scanning area of each image is 1 × 1 μm^2^

AFM scanning on the annealed alumina films produced by the two different sputtering methods (Fig. 2) revealed drastic differences in thermal stability. Figure 2a shows the AFM topographic images of the alumina films prepared by a sputtering process with argon plasma only, whereas the images of Fig. 2b were obtained from the alumina films sputtered reactively with an argon–oxygen mixture gas. The as-deposited alumina films in Fig. 2 show very similar surface morphology. However, annealing at 850 °C produced dramatically different effects. For the non-reactively sputtered film, annealing generated many defects (approximately 180 pits/μm^2^) as shown in the second image of Fig. 2a. Here, a defect indicates the dark area in the AFM image whose height is distinctly lower than the intact alumina surface. The measured depths of these nanoscale defective pits were approximately 2 nm on average, and their diameters were estimated to be 10–50 nm wide from the AFM topology. The root mean square (RMS) roughness of the defective alumina film was 0.5 nm. Fe/Mo/alumina catalyst layers also showed an inhomogeneous surface after annealing, apparently resulting from the unstable alumina underlayer. The surface presented intact areas as well as highly sintered ones in which the catalyst nanoparticles were hardly distinguishable.

In contrast, the oxygen-assisted reactive sputtering dramatically improved thermal stability, and the alumina maintained a smoother, defect-free surface after annealing in the same condition (Fig. 2b). The RMS roughness of the annealed alumina was significantly reduced to 0.2 nm. Fe/Mo ad-layers also formed well-defined sub-2-nm nanoparticles (in height) on the alumina layer (Additional file 1: Figure S3). Based on these findings, we use the terms *unstable* and *stable alumina film* in this report to denote the alumina film sputtered with argon only and with argon and oxygen, respectively.

The thermal stability of alumina thin films has been investigated previously in relation to fabrication of complementary metal oxide semiconductor (CMOS) devices. In these studies, very thin alumina layers (1–5 nm thick) on Si (001) ruptured or generated pinholes at high temperatures (900–1000 °C) under ultrahigh-vacuum (UHV) conditions [23, 24]. It was suggested that the formation of volatile species (Al_2_O, AlO, Al, O, SiO, etc.) and the subsequent desorption were the causes of the observed thermal instability [23, 25, 26]. Despite the relatively lower annealing temperature (850 °C), our annealed alumina films display a great morphological resemblance with those presented in these precedent studies. Therefore, we argue that the defect formation in our unstable films can also be related to desorption of such volatile alumina species (AlO_*x*_, *x* < 1.5) and reduced silicon oxides (SiO). Moreover, the presence of a reducing atmosphere (40 vol% of hydrogen) and the longer annealing time in our process can facilitate such volatilization.

In other studies, annealing (or growth) processes did not generate apparent film defects from alumina films prepared by a regular sputtering method [13, 16]. We speculate that this discrepancy originates from the relatively lower process temperatures of those studies (*T* < 750 °C) compared with 850 °C of our work. Indeed, the defect formation of our unstable alumina film was remarkably suppressed at 750 °C (see Additional file 1: Figure S1). In addition, the above studies used growth-promoting oxidizers such as water vapor that possibly induced chemical modification of alumina during the annealing process.

### Composition of Sputtered Alumina Layer

Our experimental findings point to a dramatic change in alumina thermal stability due to the introduction of oxygen during the sputtering process. Recently, Ohashi et al. reported that alumina films prepared by sputtering are more stable at the growth temperature of single-walled CNTs than those deposited by a thermal evaporator [18]. Their X-ray photoelectron spectroscopy (XPS) inspection revealed higher oxygen contents at the surface of the stable alumina, and growth of single-walled CNTs was highly preferred on the stable alumina layer. In contrast, their unstable alumina contained metallic aluminum domains, which was suggested as the main cause of thermal instability.

Whereas Ohashi et al. prepared alumina by exposing metallic aluminum layers to the ambient air, the alumina films in our study were prepared by sputtering a ceramic alumina target. Thus, the existence of a metallic domain in the film is unlikely (see Additional file 1: Figure S2). Instead, the introduction of oxygen gas during sputtering could augment the film oxygen content. Because the surface of our alumina films was also exposed to the ambient air after the sputtering process, the film surface could have been further oxidized by ambient air and moisture, possibly forming aluminum hydroxide whose Al/O ratio (> 2) is higher than that of the stoichiometric alumina (1.5) [18]. Therefore, to probe the possible change in composition due only to oxygen addition during sputtering, we first prepared a cross-section of the alumina layer by argon ion milling and then analyzed the film bulk by TEM and EDX.

**Fig. 3:**
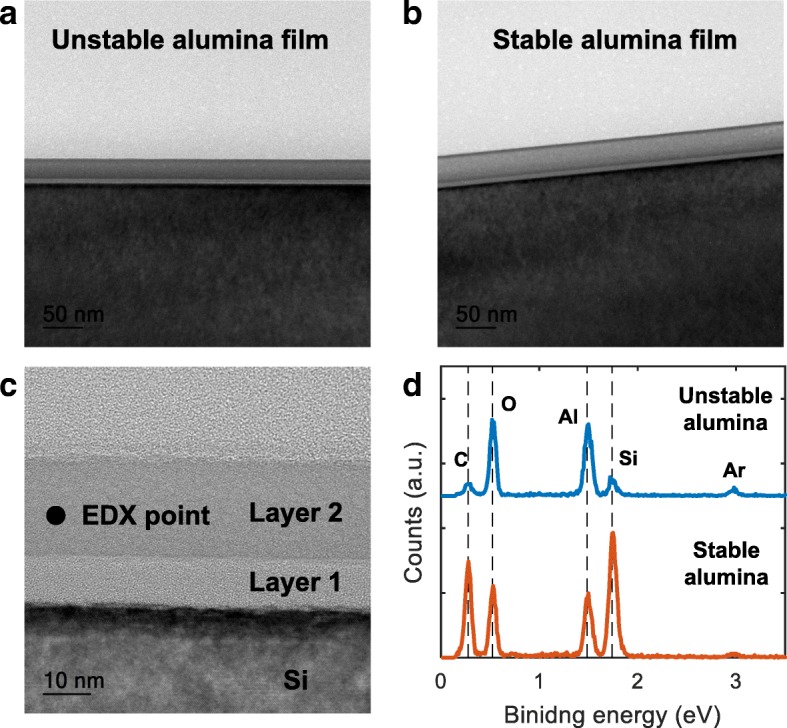
**a** Cross-sectional TEM images of the unstable alumina film. **b** Cross-sectional TEM images of the stable alumina film. **c** High-resolution TEM image of the unstable alumina film that shows two different layers of the alumina film. **d** EDX spectra detected from the middle of the film cross-section

Figure 3a, b shows the cross-sectional images of the unstable and stable alumina films. Interestingly, in both types of alumina, a bright intermediate layer is visible at the interface with the silicon substrate. This is more obvious in the high-resolution TEM image of Fig. 3c (layer 1). This intermediate layer (denoted by layer 1 in the high-resolution TEM image of Fig. 3c) is likely related to the formation of aluminum silicate during deposition of aluminum oxide on silicon, which was reported by multiple studies [20, 27]. Nayar et al. especially showed that an aluminum silicate can form on a Si wafer by electron-beam evaporation of alumina even without heating the Si substrate [20]. They suggested that silicon atoms diffuse from the underlying substrate to the growing film and react with a trace amount of water existing inside the deposition chamber. Because the base pressure of our sputtering environment was close to theirs (3–7 mPa), a similar mechanism could be responsible for the formation of the intermediate layer 1.

Despite the common formation of the intermediate layer (layer 1) in the unstable and stable films, cross-sectional EDX analysis of layer 2 (Fig. 3d) reveals a stark difference in composition of our unstable and stable alumina films. Table 1 summarizes the calculated O/Al and Si/Al atomic ratios based on the EDX spectra and shows that, although the relative oxygen content is only slightly higher, the Si/Al atomic ratio is almost 10 times greater in the stable alumina. This finding strongly suggests that the diffusion of silicon was dramatically promoted under the oxygen-rich sputtering atmosphere, leading to enhanced thermal stability.

**Table 1:** Calculated O/Al and Si/Al ratios based on atomic percentages measured by EDX

Film	O/Al ratio	Si/Al ratio
Unstable alumina	2.26	0.24
Stable alumina	2.43	2.22

We argue that the high Si content in layer 2 is responsible for the enhanced thermal stability of stable alumina films. Our claim is informed by and agrees with Bolvardi et al.’s study [19], which demonstrated that a thermal stability window of the Si-alloyed alumina film is more than 100 °C wider than that of a pure alumina. Using density-functional theory (DFT) molecular dynamics simulations, the same authors proved that the enhanced thermal stability is due to the higher strength of the Si-O bond with respect to the Al-O bond. In a similar manner, our Si-rich Si-Al-O alloy films would benefit from an increased number of Si-O bonds, resulting in dramatically improved thermal stability at 850 °C. Note also that the upper boundary of the thermal stability window corresponds to the occurrence of a phase change, and the atomic rearrangements for this phase transition are likely the source of the defects seen in our unstable alumina by AFM analysis.

### CVD Growth of VA-SWCNTs

VA-SWCNTs were produced from the prepared Fe/Mo/alumina catalysts at 850 °C. Lowering the growth temperature mitigated defect formation in the alumina films, but the growth yield of SWCNTs was also significantly reduced. Since we used a hot-wall reactor, we attribute this low growth yield to retarded gas phase reactions of the ethylene gas at a lower temperature [28]. Zhong et al. [29] also consistently demonstrated that a higher concentration of active carbon precursor gases increased the growth yield of VA-SWCNTs, possibly owing to enhanced nucleation in carbon-rich conditions.

**Fig. 4:**
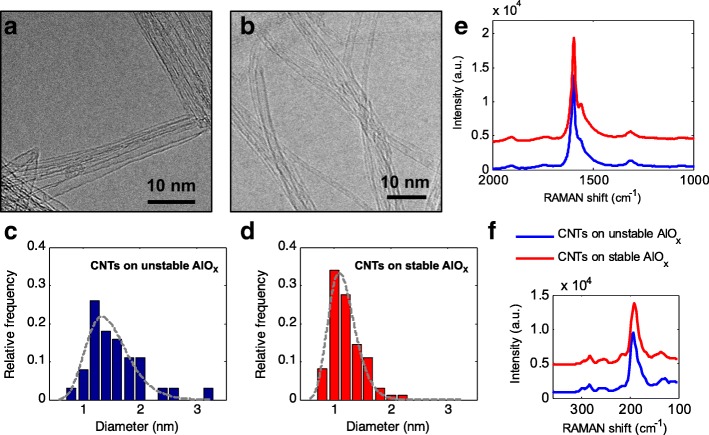
**a**, **b** TEM images of CNTs grown on alumina deposited by **a** nonreactive sputtering and **b** reactive sputtering. CNTs grew from the catalyst with different diameter distributions; histograms (**c**) and (**d**) result from analysis of many images such as (**a**) and (**b**), respectively. The mean diameters of **c** and **d** are approximately 1.4 and 1.2 nm, respectively. **e**, **f** Raman spectra (excitation at 632.8 nm) collected from the top of the produced nanotube forests. The red (upper) curve indicates the Raman spectrum from CNTs on the stable alumina, and the blue (lower) curve corresponds to the spectrum from CNTs on the unstable alumina

TEM images in Fig. 4a, b confirm growth of SWCNTs from the catalyst layers. The diameter distribution of the as-grown VA-SWCNTs (Fig. 4c, d) was determined from similar TEM images. Although VA-SWNTs also grew successfully on the unstable alumina layer, their distribution (mean 1.4 nm, SD 0.5 nm) was shifted to larger diameters and was slightly broader compared to that of VA-SWNTs from stable alumina (mean 1.2 nm, SD 0.4 nm). In both cases, the diameter distribution can be fitted to a lognormal function (dashed lines in Fig. 4c, d), which is skewed toward smaller diameters [29].

The Raman spectra of both CNT forests (Fig. 4e) look similar; however, the shoulder peak (~ 1570 cm^−1^) in the G-band (at ~ 1595 cm^−1^), which is a typical characteristic of SWCNTs, is more defined in the CNTs grown on the stable alumina support. The G/D ratios are close to 10 in both cases, indicating high quality of the grown CNT arrays. The high intensity of the radial breathing modes (peaks at 150–300 cm^−1^) confirms abundant presence of sub-2-nm-wide CNTs.

In addition, growths from stable and unstable supporting layers differed in terms of CNT length and reproducibility. When compared with stable alumina, growth of VA-SWCNTs from unstable alumina terminated earlier during the CVD process and produced shorter CNTs. The growth termination time was also unpredictable. The earlier growth termination can be explained with a more significant subsurface diffusion of Fe/Mo catalyst particles, promoted by the instability of the underlying alumina layer. This argument agrees with Tsuji et al.’s result [17]. They suggested that growth of VA-SWCNTs can be significantly extended by thermally healing structural defects of the supporting layer and thereby retarding subsurface diffusion.

### Ion Transport Through SWCNT Membranes

In our previous studies [6, 30], we demonstrated that membranes with small-diameter VA-SWCNTs as the only pores enable selective permeation of ions while maintaining very high water fluxes. The observed rejection for small ions was due to electrostatic interactions between the ions in solutions and charged carboxylic groups at the SWCNT tip formed during nanotube opening in an oxidizing atmosphere [6]. Ion selectivity followed Donnan theory in a semiquantitative way. For small ions such as potassium, chloride, and sulfate, size exclusion or hydrodynamic interaction did not play a significant role [6], likely because the hydrated ion size was small enough to fit into the smallest CNTs of the previously produced membranes and because of the smoothness of the inner SWCNT graphitic walls. Even when electrostatic interactions dominate the rejection mechanism, membrane selectivity is expected to be sensitive to pore diameter and detrimentally affected by the presence of a tail of large diameter pores. Indeed, in the same solution conditions, electrostatic interactions work more efficiently at excluding anions from narrower pores because, in the same solution conditions, the ratio between Debye length and pore diameter becomes larger. In other words, the distance from the CNT rim to the pore center that needs to be bridged by the electrostatic forces to “close” the pore is shorter for smaller-diameter pores [6]. Because of the narrower pore size distribution and the shift to small diameters, stable alumina supports are expected to enable fabrication of membranes with enhanced ion rejection properties. Moreover, reducing the pore diameters may allow entering a transport regime where size exclusion plays a nonnegligible role in determining the overall membrane selectivity.

**Fig. 5:**
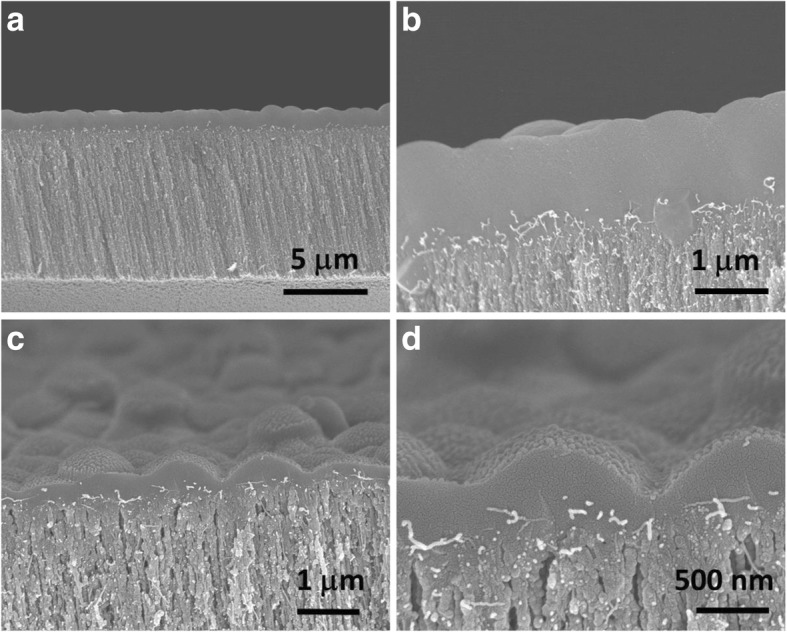
Cross-sectional SEM images of CNT-SiN_x_ membranes. **a** Low- and **b** high-magnification images of a CNT membrane before opening the CNT pores with etching steps and, thus, before ion rejection studies. **c** Low- and **d** high-magnification images of the top surface of a CNT membrane after etching and after ion filtration studies. In all images, a dense SiN_x_ layer on the membrane surface and the vertical alignments of the CNTs in the composite are clearly visible. After etching, CNT bundles emerge from the membrane top surface

**Fig. 6:**
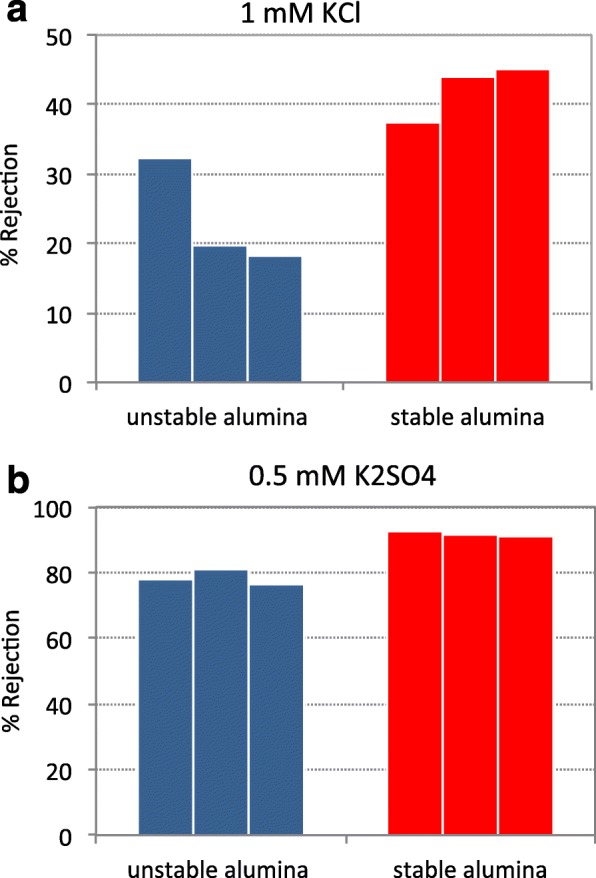
Anion rejection coefficient for three membranes fabricated with VA-SWCNTs grown on stable (red) and unstable alumina (blue): **a** filtration of a 1 mM KCl solution; **b** filtration of a 0.5 mM K_2_SO_4_ solution. % Rejection = [1 − (*c*_permeate_/*c*_feed_)] × 100, where *c*_permeate_ and *c*_feed_ are the ion concentrations in permeate and feed, respectively

To verify our claim, we fabricated membranes with VA-SWCNT arrays grown on both unstable and stable alumina films and compared the ion rejection performance of these membranes for two salt solutions (1 mM KCl and 0.5 mM K_2_SO_4_) under the same experimental conditions (see Fig. 5 for cross-sectional images of membranes before and after filtration tests). In both cases, we calculated the rejection coefficient for three membranes as 1 − (*c*_permeate_/*c*_feed_). The results reported in Fig. 6 unambiguously reveal that the shift to smaller SWCNT diameters (both average and maximum) translated into a 15–20% and ~ 12% increase in KCl and K_2_SO_4_ rejection coefficients, respectively. No dedicated experiments were performed to decouple the contribution of the two mechanisms (size exclusion and electrostatic interaction) to the ion selectivity of the SWCNTs grown on stable alumina. However, because the hydrated radius of the largest anion (sulfate) was only 0.379 nm and the largest rejection enhancement was obtained for the smallest anion, the recorded improvement of the rejection performance can likely be attributed to a more efficient electrostatic exclusion rather than to size effects.

## Conclusions

In summary, our results show (a) a stark improvement in the thermal stability of alumina films sputtered in an oxygen-containing atmosphere, (b) a narrower diameter distribution for the SWCNTs grown on the thermally stable alumina layer, and (c) a correspondingly higher ion selectivity for the membranes fabricated with these SWCNTs. High-temperature annealing at 850 °C induces defective pits in alumina support layers sputtered without oxygen gas. Conversely, oxygen-reactive sputtering promotes the formation of Si-rich alumina layers with higher thermal stability. This stable support favors reliable growth of narrowly distributed sub-2-nm VA-SWCNTs. Nanofiltration membranes made from these VA-SWCNTs display improved ion rejection in pressure-driving filtration experiments thanks to the smaller diameters of these CNT arrays. Our reactive sputtering method could be combined with post-treatment techniques such as ambient annealing [17], oxygen plasma treatment [31], and ion beam bombardment [32] to further enhance the stability of supporting layers.

## Additional file

Additional file 1: **Figure S1.** AFM on an unstable alumina film annealed at 750 °C. **Figure S2.** Al 2p XPS spectra of the unstable and the stable alumina films. **Figure S3.** Diameter distribution of the catalyst particles after annealing extracted from AFM image analysis (bars). Top: unstable alumina films; Bottom: stable alumina films. Overlapping dashed curves represent the corresponding SWCNT diameter distribution measured by TEM. **Table S1.** Typical methods used in the literature to verify that transport occurs through CNTs rather than through defects in the matrix. NP = nanoparticle; *P*_*gas*_ = gas permeance. (DOCX 459 kb)

## Abbreviations

AFM: Atomic force microscopy
CNT: Carbon nanotube
CVD: Chemical vapor deposition
EDX: Energy-dispersive X-ray spectroscopy
RMS: Root mean square
SD: Standard deviation
SWCNT: Single-walled carbon nanotube
TEM: Transmission electron microscopy
VA-SWCNT: Vertically aligned single-walled carbon nanotube
