# Supplementary material for: Effect of Enhanced Thermal Stability of Alumina Support Layer on Growth of Vertically Aligned Single-Walled Carbon Nanotubes and Their Application in Nanofiltration Membranes
Source: Nanoscale Res Lett. 2018 Jun 7;13:173. doi: 10.1186/s11671-018-2585-3 (PMC5992115; doi:10.1186/s11671-018-2585-3)
Supplement: Supplementary file 1 — Figure S1. AFM on an unstable alumina film annealed at 750 °C. Figure S2. Al 2p XPS spectra of the unstable and the stable alumina films. Figure S3. Diameter distribution of the catalyst particles after annealing extracted from AFM image analysis (bars). Top: unstable alumina films; Bottom: stable alumina films. Overlapping dashed curves represent the corresponding SWCNT diameter distribution measured by TEM. Table S1. Typical methods used in the literature to verify that transport occurs through CNTs rather than through defects in the matrix. NP = nanoparticle; Pgas = gas permeance. (DOCX 459 kb) [file 11671_2018_2585_MOESM1_ESM.docx]

## Additional file 1


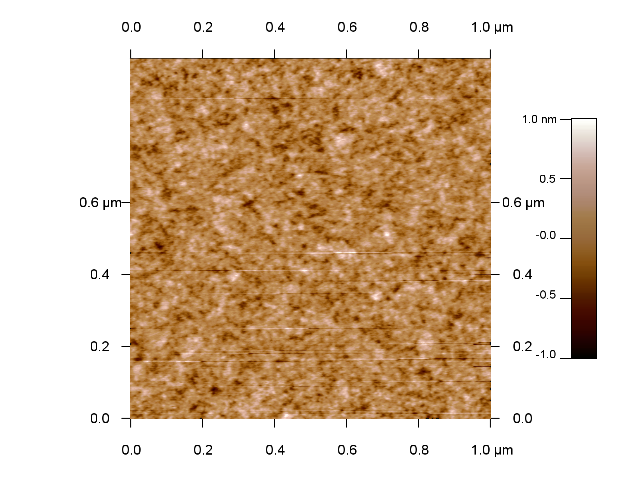


Figure S1. AFM on an unstable alumina film annealed at 750 °C


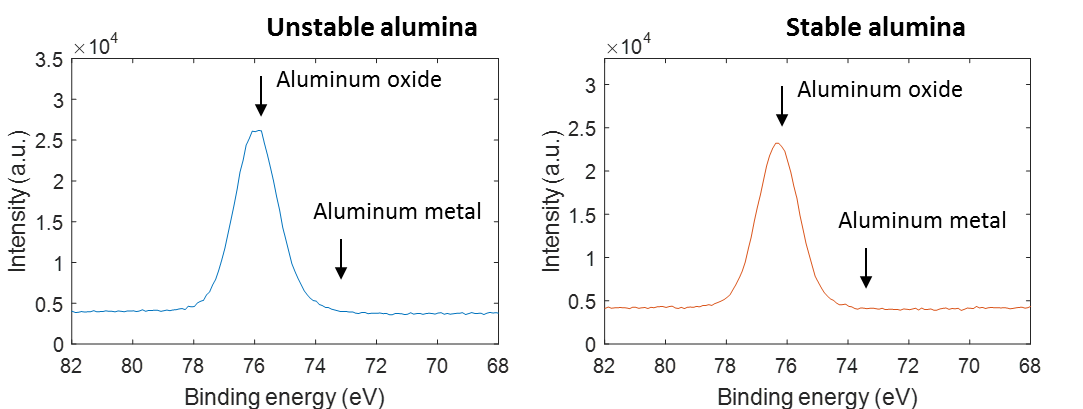


Figure S2. Al 2p XPS spectra of the unstable and the stable alumina films

The diameter of CNTs is expected to be closely related to the size of Fe/Mo catalyst particles formed during growth. We compared here the diameter distribution of the catalyst particles after annealing extracted from AFM image analysis with those of our SWCNTs measured by TEM (Figure S3). Note that, in the case of the annealed “metal-deposited alumina” of Fig. 2a, the background topology (alumina before metal deposition) is quite rough and its pre-existing topological features are similar in height to the Fe/Mo catalyst particles. Moreover, the metal catalysts on unstable alumina appear to form highly sintered area where individual particles are not easily distinguishable. Therefore, a regular height analysis of the AFM topology data is not expected to be accurate for the unstable alumina case. Regardless, Figure S3 shows a good correlation between the distribution of catalyst particle sizes and CNT diameters, as well as between their shift with alumina stability.


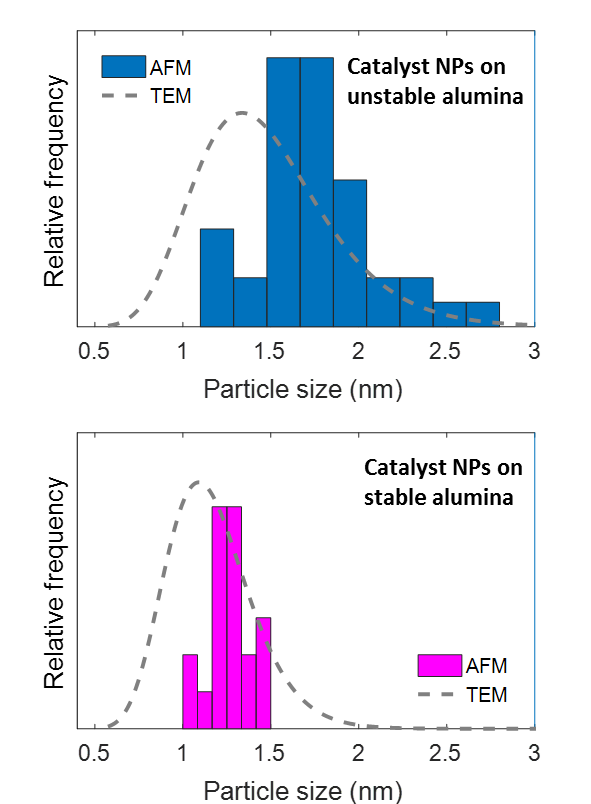


Figure S3. Diameter distribution of the catalyst particles after annealing extracted from AFM image analysis (bars). Top: unstable alumina films; Bottom: stable alumina films. Overlapping dashed curves represent the corresponding SWCNT diameter distribution measured by TEM.

Table S1. Typical methods used in the literature to verify that transport occurs through CNTs rather than through defects in the matrix. NP = nanoparticle; *P_gas_* = gas permeance.

| References | No flow before etching steps to open CNTs | Imaging of membrane cross-section | P_gas_ pressure independent and/or Knudsen selectivity | PEG, protein, NP filtration | Ion selectivity (ion rejection or current rectification) | Selectivity change with CNT tip chemistry |
| --- | --- | --- | --- | --- | --- | --- |
| Zhang [[1](#_ENREF_1)] | yes | yes | yes | - | - | - |
| Park [[2](#_ENREF_2)] | - | - | - | yes | yes | - |
| Krishnakumar [[3](#_ENREF_3)] | yes | - | - | yes | yes | yes |
| Mi [[4](#_ENREF_4)] | - | yes | yes | - | - | - |
| Zhang [[5](#_ENREF_5)] | - | yes | yes | - | - | - |
| Holt [[6](#_ENREF_6)] | yes | yes | yes | yes | - | - |
| Baek [[7](#_ENREF_7)] | yes | yes | - | - | - | - |
| Lee [[8](#_ENREF_8)]* | yes | yes | - | yes*** | - | - |
| Du [[9](#_ENREF_9)]* | - | - | - | yes | - | - |
| Majumder [[10](#_ENREF_10)] | yes | yes | - | yes | - | yes |
| Majumder [[11](#_ENREF_11)]** | yes | yes | yes | - | - | - |
| Wu [[12](#_ENREF_12)] | yes | yes | - | - | yes | - |
| Kim [[13](#_ENREF_13)] | yes | yes | yes | yes | yes | - |
| Fornasiero [[14](#_ENREF_14), [15](#_ENREF_15)] | yes | yes | yes | yes | yes | - |
| Bui [[16](#_ENREF_16)] | yes | yes | yes | yes | yes | - |
| This work | yes | yes | yes | yes | yes | - |

Additional supporting result:

* dye mixed in the polymer matrix was not detected in the permeate, suggesting no liquid flow through the matrix

** functionalization of the inner CNT wall resulted in a loss of ultrafast water flow

*** pore size distribution extracted from PEG rejection studies match CNT diameter distribution from TEM

**References**

[1] Zhang L, Yang J, Wang X, Zhao B, Zheng G. Temperature-dependent gas transport performance of vertically aligned carbon nanotube/parylene composite membranes. Nanoscale Res Lett. 2014;9.

[2] Park S-M, Jung J, Lee S, Baek Y, Yoon J, Seo DK, et al. Fouling and rejection behavior of carbon nanotube membranes. Desalination. 2014;343:180-6.

[3] Krishnakumar P, Tiwari PB, Staples S, Luo T, Darici Y, He J, et al. Mass transport through vertically aligned large diameter MWCNTs embedded in parylene. Nanotechnol. 2012;23(45):455101.

[4] Mi WL, Lin YS, Li YD. Vertically aligned carbon nanotube membranes on macroporous alumina supports. J Membr Sci. 2007;304:1-7.

[5] Zhang L, Zhao B, Wang X, Liang Y, Qiu H, Zheng G, et al. Gas transport in vertically-aligned carbon nanotube/parylene composite membranes. Carbon. 2014;66(0):11-7.

[6] Holt JK, Park HG, Wang YM, Stadermann M, Artyukhin AB, Grigoropoulos CP, et al. Fast mass transport through sub-2-nanometer carbon nanotubes. Science. 2006;312(5776):1034-7.

[7] Baek Y, Kim C, Seo DK, Kim T, Lee JS, Kim YH, et al. High performance and antifouling vertically aligned carbon nanotube membrane for water purification. J Membr Sci. 2014;460:171-7.

[8] Lee K-J, Park H-D. The most densified vertically-aligned carbon nanotube membranes and their normalized water permeability and high pressure durability. J Membr Sci. 2016;501:144-51.

[9] Du F, Qu LT, Xia ZH, Feng LF, Dai LM. Membranes of Vertically Aligned Superlong Carbon Nanotubes. Langmuir. 2011;27(13):8437-43.

[10] Majumder M, Chopra N, Andrews R, Hinds BJ. Nanoscale hydrodynamics - Enhanced flow in carbon nanotubes. Nature. 2005;438(7064):44.

[11] Majumder M, Chopra N, Hinds BJ. Mass transport through carbon nanotube membranes in three different regimes: ionic diffusion and gas and liquid flow. ACS Nano. 2011;5(5):3867-77.

[12] Wu J, Gerstandt K, Zhang H, Liu J, Hinds BJ. Electrophoretically induced aqueous flow through single-walled carbon nanotube membranes. Nat Nano. 2012;7:133-9.

[13] Kim S, Fornasiero F, Park HG, Bin In J, Meshot E, Giraldo G, et al. Fabrication of flexible, aligned carbon nanotube/polymer composite membranes by in-situ polymerization. J Membr Sci. 2014;460:91-8.

[14] Fornasiero F, Bin In J, Kim S, Park HG, Wang Y, Grigoropoulos CP, et al. pH-tunable ion selectivity in carbon nanotube pores. Langmuir. 2010;26(18):14848-53.

[15] Fornasiero F, Park HG, Holt JK, Stadermann M, Grigoropoulos CP, Noy A, et al. Ion exclusion by sub-2-nm carbon nanotube pores. Proc Natl Acad Sci U S A. 2008;105(45):17250-5.

[16] Bui N, Meshot ER, Kim S, Peña J, Gibson PW, Wu KJ, et al. Ultrabreathable and Protective Membranes with Sub-5 nm Carbon Nanotube Pores. Adv Mater. 2016;28(28):5871-7.
